# Improvement of blood transfusion safety using the chemiluminescence technique for viral marker screening of blood donors in sub Saharan Africa

**DOI:** 10.1016/j.htct.2024.04.120

**Published:** 2024-09-07

**Authors:** Macoura GADJI, Aissata BA, Youssou Bamar GUEYE, Alioune Badara SENGHOR, Tandakha Ndiaye DIEYE, Saliou DIOP

**Affiliations:** a Service of Biological Haematology & Oncology-Haematology (BHOH), Department of Biology and Applied Pharmaceutical Sciences, Faculty of Medicine, Pharmacy and Odonto-Stomatology (FMPOS), University Cheikh Anta Diop of Dakar (UCAD), Dakar, Senegal; b National Centre of Blood Transfusion (NCBT/CNTS), Dakar, Senegal; c Service of Immunology; Department of Biology and Applied Pharmaceutical Sciences; Faculty of Medicine, Pharmacy and Odonto-Stomatology (FMPOS), University Cheikh Anta Diop of Dakar (UCAD), Dakar, Senegal; d Service of Haematology; Department of Medicine; Faculty of Medicine, Pharmacy and Odonto-Stomatology (FMPOS), University Cheikh Anta Diop of Dakar (UCAD), Dakar, Senegal

**Keywords:** Blood transfusion, Transfusion-transmitted infectious agents, Blood safety, Chemiluminescence, Immunoenzymatic, Immunochromatographic, Sub-Saharan Africa

## Abstract

**Introduction:**

Sub-Saharan Africa struggles continuously with insufficient resources and inadequate infrastructure that hinder the establishment of a safer blood supply despite improvements in transfusion safety over recent decades. This study aimed to evaluate the impact of the chemiluminescence technique in combination with immunoenzymatic and immunochromatographic tests for viral marker screening of hepatitis B (HBV), hepatitis C (HCV) and human immunodeficiency virus (HIV) in donated blood in a country of sub-Saharan Africa.

**Method:**

This study was conducted in a population of 113,406 blood donors at the National Centre of Blood Transfusion in Senegal. The data were obtained from the ‘INLOG’ software and donor registers. Statistical analyses used Excel 2010 and Epi Info v6. Screening for HBsAg viral markers, anti-HCV Ab, HIV p24 Ag, anti-HIV1 and anti-HIV2 antibodies were first carried out using the chemiluminescence technique. Blood donations screened positive for HBV or HCV were retested in a second chemiluminescence equipment. HIV-positive donations and their controls were subjected to solid phase immunochromatographic and indirect enzyme immunoassay techniques.

**Results:**

The prevalence among donors of HBV was 8.39 %, 0.56 % for HCV and 0.18 % for HIV. Of the donors tested positive for HIV in screenings and in doubled-controls, only 61.54 % were confirmed by the alternative tests; 34.02 % were negative and 4.44 % discordant between the three techniques.

**Conclusion:**

This study shows the importance of introducing the chemiluminescence technique in association with serological screening of transfusion-transmitted viruses to improve blood supply safety in low-income countries.

## Introduction

Screening for infectious agents constitutes a very critical step in the process of biological qualification of donated blood. It helps prevent transfusion-transmitted infections (TTIs) through blood and its derivatives.^1^ Despite important improvements in transfusion safety over the past decades, safety of the blood supply remains a public health problem in low- and middle-income countries (LMICs). Indeed, these economically restricted countries continue to struggle with inadequate infrastructure and lack of economic and political resources that hinder the establishment of a safer blood supply.^2^^,^^3^ According to the World Health Organization (WHO), of the 118.5 million blood donations collected globally in 2018, 40 % were collected in high-income countries (HICs), home to 16 % of the world's population (WHO/fact-sheets/blood-safety-and-availability in March 2022). Moreover, approximately 80 % of the world's population resides in LMICs and benefits from only 20 % of the worldwide supply of safe blood.^2^^,^^3^ Despite differences in disease epidemiology and financial resources between countries (LMICs and HICs), the WHO strategy for laboratory screening of blood recommends HIV, HBV, and syphilis screening of all donated blood and, when necessary or appropriate, screening for HCV, malaria, and Chagas disease.^4^ Notwithstanding, the lack of economic and political resources has compromised the blood supply in many areas of the world, in particular, in sub-Saharan Africa (SSA).

However, although the WHO standard has not yet been achieved, much progress has been made in SSA. Indeed, the safety of blood products has improved steadily in recent years thanks to better donor selection^5^^,^^6^ and concomitant to the implementation of increasingly efficient screening techniques. In SSA, the prevalence of infectious agents is high in the general population, particularly HBV, HCV and HIV.^7^^,^^8^ These high prevalence rates increase the risk of transmission through blood transfusion.^9^^,^^10^ Blood transfusion is one of the most widely used therapies in Africa countries south of the Sahara.^4^, ^11^ Despite the implementation of rules and measures related to the clinical selection of blood donors and screening for infectious diseases in donated blood, the risk of transmission of a virus or bacteria during blood transfusion remains critical in these countries.^4^, ^11^ This risk may be linked to infectious donations collected during the window of immunosilence, to viral variants, the antibodies of which are not recognized by current serological tests, and to viremic subjects who remain seronegative over a longer period. Indeed, the possibility of chronic carriage without detectable antibodies has been described in immunocompromised patients.^12^ Transfusion safety, based on controlling the immunological risk and reducing transmissible infections^5^ is a major public health issue in the world, more particularly in developing countries. The possibilities of the early diagnosis of viruses transmitted by blood or its derivatives should therefore be constantly sought. Significant progress has thus been made in the screening of blood donations by the development of increasingly efficient techniques, namely: enzyme-linked immunosorbent assay (ELISA) on microplates, rapid serological tests, automated systems based on immunochromatography, enzyme immunoassay (EIA) and chemiluminescence.

Different approaches using chemiluminescence were developed but particle-based chemiluminescence immunoassay technology is the most conventional, including direct chemiluminescence immunoassay (CLIA), chemiluminescence enzyme immunoassay (CLEIA), electrochemiluminescence immunoassay (ECLIA) and photo-induced chemiluminescence immunoassay (LiCA).^13^, ^14^, ^15^, ^16^, ^17^ In general, screening tests for HBsAg, and HIV and HCV antibodies are performed with EIA test kits worldwide however rapid serological tests are still performed in several countries in SSA. All these techniques have been chronologically used at the National Centre of Blood Transfusion in Senegal (NCBT) though this ended five years ago with the introduction of the chemiluminescence technique using magnetic particles.

This study aimed to evaluate the use of this chemiluminescence technique in combination with immunochromatography and EIA in the screening of infectious viral agents (HBV, HCV and HIV) in donated blood while updating the frequencies of these viruses in blood donors in a country in the southern Saharan region.

## Method

### Study type and donor population

A retrospective study on the first four years of the utilization of the chemiluminescence technique for screening viral agents in donated blood was conducted at the NCBT.^13^^,^^16^, ^17^, ^18^, ^19^ The studied population consisted of unpaid voluntary blood donors who met the eligibility criteria for donating blood in accordance with the general rules for transfusion safety established by the WHO.^20^^,^^21^ Suitability or inability to donate blood was determined by the collecting physician as previously reported.^5^^,^^6^ Insufficiently filled blood bags were excluded from the transfusion process.

### Collecting blood samples

Blood samples were collected in dry tubes or tubes containing ethylenediaminetetraacetic acid (EDTA) from the bags at the time of blood donation. These samples were centrifuged at 3000 rpm for seven minutes to obtain serum or plasma which was used for screening for viral agents.

### Screening for viral agents

Chemiluminescence is a phenomenon of light radiation accompanying substances in the process of chemical reactions. Combining chemiluminescence detection technology with immune response forms a chemiluminescence immunoassay (CMIA) technology, which can be divided into microplate and microparticle according to different fixed carrier phases.^13^^,^^15^, ^16^, ^17^^,^^19^

This study used the ARCHITECT *i1000SR* immunoassay analyzer (Abbott, USA) which operates according to the chemiluminescence technique to detect classic Ag-Ab reactions on the surface of magnetic microparticles followed by an electrochemical reaction on the surface of an electrode resulting in measurable and quantifiable light emission. CMIA was used only to screen for HBsAg (Architect HBs Ag Qualitative II Reagent Kit/2G22) and anti-HCV Ab (Architect Anti-HCV/Kit 6C37) in each sample. The Architect HIV Ag/Ab Combo Reagent Kit/4j27 was used to screen for HIV p24 Ag, anti-HIV1 and anti-HIV2 antibodies. If a sample was positive by chemiluminescence, the test was repeated in duplicate in another series of tests with a second Architect *i1000SR* equipment. Of course, if one or both of the repeat assays was positive, the sample was considered positive, the donation was discarded and the donor deferred. Each doubly positive HIV sample (first screening and second duplicate screening) was then tested by two other techniques; an Alere Determine™ HIV1/2 immunochromatographic test [Test Alere Determine™ HIV-1/2 Serum/Plasma, (Ref, 7D2342) or (Ref 7D2343) (Alere, France)] and an Immunocomb II HIV1 and 2 BiSpot immunoenzymatic tests (Orgenics, France) (Figure S1). According to the manufacturer's guidelines, the Architect HIV Ag/Ab Combo has a sensitivity of 100 % and a specificity ≥ 99.5 % for blood donor HIV screening however studies report values from 97 to 100 % for blood donors depending of the type of study.^22^, ^23^, ^24^

The Alere Determine HIV-1/2 test is a rapid, visual immunochromatographic test for the qualitative detection of anti-HIV1 and anti-HIV2 antibodies. On following manufacturer guidelines, Alere Determine has a sensitivity of 99.91 % and a specificity of 98.16 % in blood donor HIV screening. However, these values are reported in around 88–99 % of blood donors depending on the type of study.^25^, ^26^, ^27^, ^28^

The Immunocomb II™ HIV1 and HIV2 BiSpot test is an indirect solid phase enzyme-linked immunosorbent assay (EIA) with a sensitivity of 100 % and a specificity of 98.4 % according to the manufacturer, even though studies report lower values (90–99 %).^25^^,^^26^^,^^28^^,^^29^

### Collection of epidemiological data and statistical analyses

The data were obtained from the ‘INLOG’ software and from NCBT registers. Data were input into Excel 2010 and statistical analyses used Epi Info version 6.

## Results

### Epidemiological characteristics of blood donors

The study population consisted of 113,406 blood donors attended at the NCBT over a period of four years using the chemiluminescence technique with an annual rate of increase in the number of donors of 5.6 %. These donors were predominantly male (86,858: 76.59 %) with female donors representing 23.41 % (26,548 donors), giving a male:female ratio of 3.27. New donors (first donation) accounted for 56.46 % and regular donors (two or more blood donations) 43.53 %. The average age of donors was 35 years with a predominance of the 20- to 30-year age group.

### Prevalence of HBV, HCV and HIV viral markers

**Figure 1:**
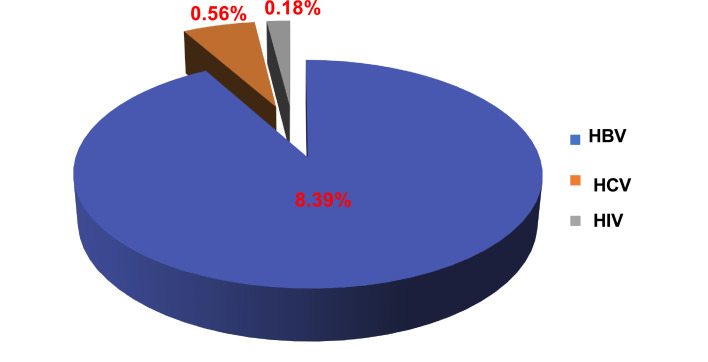
Prevalence of hepatitis B (HBV), hepatitis C (HCV) and human immunodeficiency virus (HIV) in blood donors.

Overall, seroprevalences were 8.39 % (9525 donors) for HBV, 0.56 % (634 donors) for HCV, and 0.18 % (208 donors) for HIV. Thus, out of the 113,406 blood donors, 10,367 donors or 9.13 % were positive for at least one of these markers (Figure 1). At the NCBT, all AgHBs^+^ and anti-HCV^+^ donors benefit from a clinical follow up with AgHBs or anti-HCV retesting every six months for two years. If they develop any symptoms, they are referred to the service of infectious diseases as for HIV+ donors.

### Distribution of viral markers by sex and age

**Figure 2:**
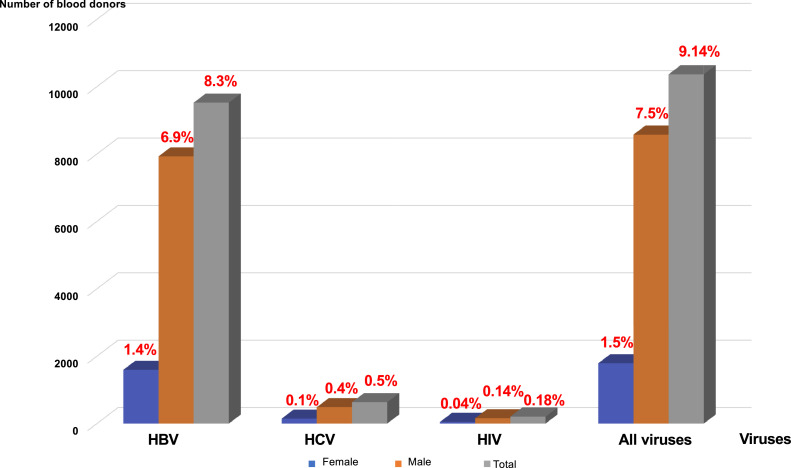
Prevalence of hepatitis B (HBV), hepatitis C (HCV) and human immunodeficiency virus (HIV) by sex.

**Figure 3:**
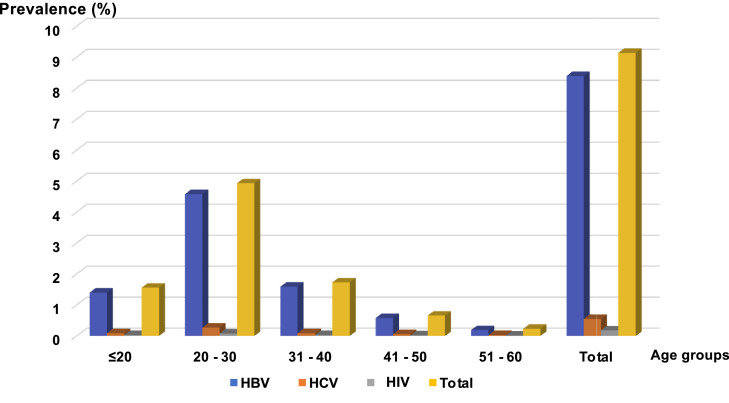
Prevalence of hepatitis B (HBV), hepatitis C (HCV) and human immunodeficiency virus (HIV) according to age group.

Male donors were the most affected: 7930 donors (6.99 %) for HBV, 487 donors (0.43 %) for HCV, and 159 donors (0.14 %) for HIV. The prevalences for female donors were 1595 donors (1.40 %) for HBV, 147 donors (0.13 %) for HCV and 49 donors (0.04 %) for HIV (Figure 2). All these prevalences were higher in the 20- to 30-year age group, followed by that of 30–40 years (18.92 %) (Figure 2, Figure 3).

### Comparison of the chemiluminescence technique with immunochromatographic and immunoenzymatic tests for HIV screening

**Figure 4:**
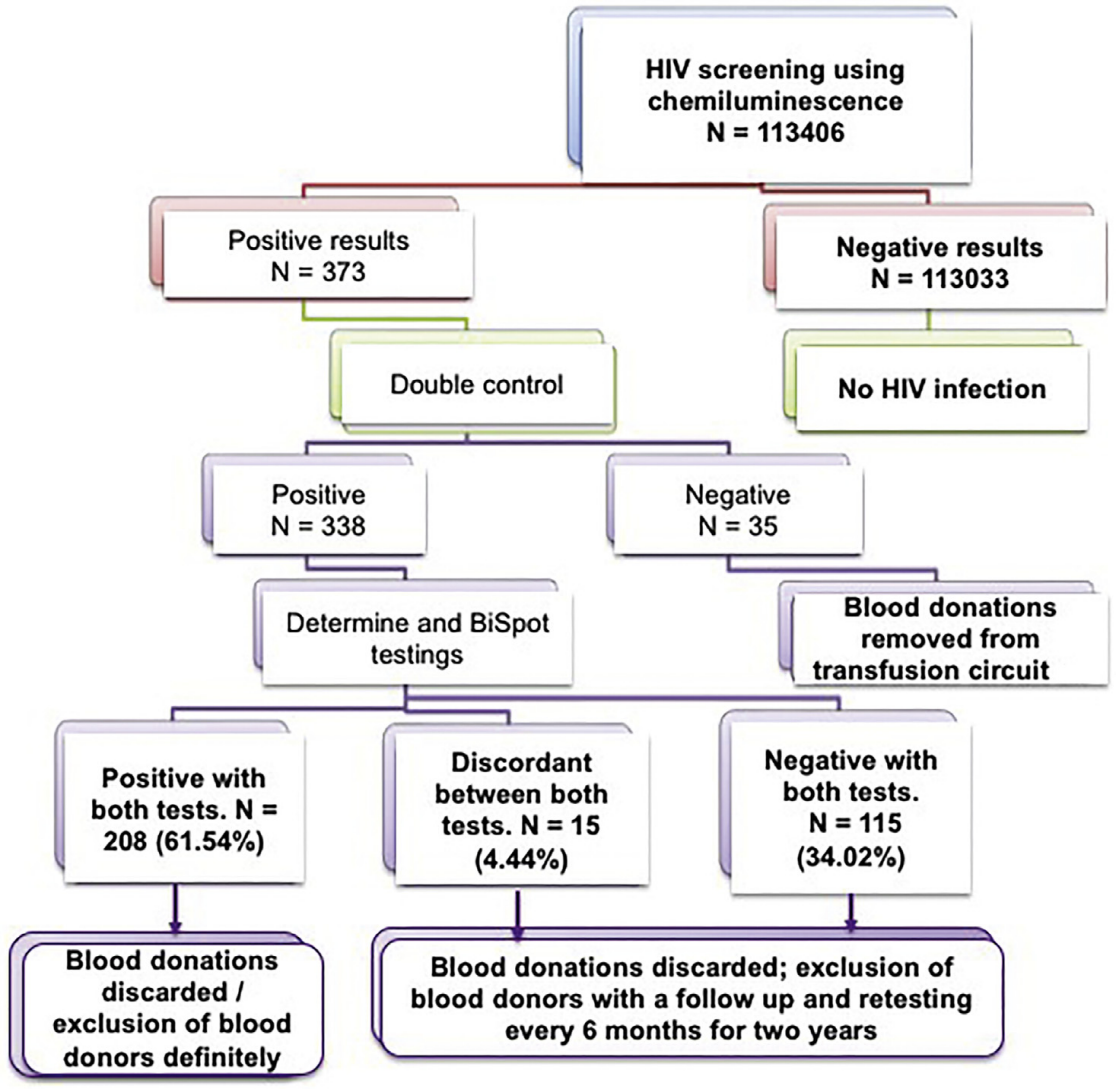
Results of immunodeficiency virus (HIV) marker screening according to our screening algorithm.

Of the 338 positive HIV samples (first and duplicate screenings) by the chemiluminescence technique, only 208 cases (61.54 %) were confirmed with both the immunoenzymatic and immunochromatographic tests, 115 cases (34.02 %) were negative by both assays and 15 cases (4.49 %) were discordant. Of the 15 discrepant cases, 9 out of 15 donors were positive by BiSpot but negative by Determine and 6 donors were positive by Determine but negative by the BiSpot test (Figure 4 and Table 1; Figure S1).

**Table 1:** Discordant results by chemiluminescence technique with immunochromatographic and immunoenzymatic techniques for the detection of viral markers of HIV in blood donors.

Blood donors ID	Chemiluminescence	URL	immunochromato- graphic	Immunoenzymatic
1	Positive	1.23	Positive	Negative
2	Positive	1.88	Positive	Negative
3	Positive	2.28	Positive	Negative
4	Positive	2.66	Positive	Negative
5	Positive	1.25	Positive	Negative
6	Positive	2.82	Positive	Negative
7	Positive	1.17	Negative	HIV1 Positive
8	Positive	1.23	Negative	HIV1 Positive
9	Positive	1.30	Negative	HIV1 Positive
10	Positive	1.68	Negative	HIV1 Positive
11	Positive	1.71	Negative	HIV1 Positive
12	Positive	2.51	Negative	HIV1 Positive
13	Positive	1.63	Negative	HIV1 Positive
14	Positive	1.92	Negative	HIV1 Positive
15	Positive	2.03	Negative	HIV1 Positive

## Discussion

Blood transfusion saves lives and improves health, but many patients requiring transfusion do not have timely access to safe blood worldwide, in particular in SSA. Providing a safe and adequate blood supply should be an integral part of every country's national healthcare policy and infrastructure.^20^^,^^30^ Blood transfusion policies are part of a process of continuous improvement in the quality of blood products. The goal is to have sufficient, high quality blood and blood products. The WHO recommendations published four important statements that present challenges to blood centers with financial and logistical restrictions:

• commitment of governments to a well-developed and organized blood transfusion service for a sustainable and successful blood transfusion system;
• selection of safe blood donors by recruitment of nonremunerated individuals;
• proper laboratory testing of donated blood and
• adequate record keeping

Transfusion safety relies on appropriate blood administration to recipients.^31^, ^32^, ^33^ Given the difficulties in donor screening and laboratory testing of blood, consideration must be given to balancing the risk of exposure to TTIs versus the risk of no transfusion.^31^ Appropriate screening of donated blood to avoid TTIs is mandatory for any blood transfusion service. Indeed, testing is improving as more automated and more sensitive assay systems become available and potential new infectious agents are identified. This study aimed to explore the value of introducing viral marker screenings using a chemiluminescence technique and updating the statistics on viruses linked to blood donation in a low-income country as an example of SSA countries.

The technical procedures of infectious agent screening in blood donors have developed from classical methods (ELISA) to modern automated techniques such as chemiluminescence. These latter high throughput techniques with a high level of sensitivity and specificity are more suitable for a large number of blood donors as automation reduces human interference and therefore the risk of errors. Indeed, equipped with fully automatic instruments, CLIA is simple and fast.^13^^,^^15^^,^^19^ In the last decade, CLIA has been employed frequently to detect more than one hundred items playing a very useful role in disease screening. Furthermore, CLIA has also widely used in the diagnosis of tumors, and detection of bacteria and fungi infectious, and cardiovascular, metabolic and other diseases.^14^^,^^15^^,^^18^^,^^34^ One study comparing CLIA, ELISA and lateral flow immunochromatography to detect the SARS-CoV-2 virus found sensitivities of 92 %, 86 %, and 78 %, respectively.^14^ Another study that detected antibodies in patients with vasculitis found an even higher sensitivity for CLEIA.^15^ Overall, it is considered that the Architect® system offers a specificity greater than 99.5 % in blood donor screening and a similar level in the determination of HIV p24 Ag, anti-HIV1/HIV2 antibodies and HBsAg. In anti-HCV antibody assays the specificity and sensitivity reach levels of 99.6 % and 99.1 %, respectively.^15^^,^^19^ However, in this present study, discrepancies in HIV screening were noted between the three techniques used. They occur mostly with samples with upper reference limits (URLs) near the cutoff values (Figure 4; Table 1) and may be related to anti-HIV antibodies that do not react with the specific antigens used in the immunochromatographic assay setup or due to infections with a variant of the virus difficult to detect by the configuration of the enzyme immunoassay.^15^^,^^24^, ^25^, ^26^, ^27^, ^28^, ^29^^,^^35^ The high sensitivity of the chemiluminescence technique may be responsible for these false positives, resulting in a reduction not only in the number of available blood products but also in the number of blood donors. Moreover, the chemiluminescence technique, due to its high sensitivity and automation, ensures high transfusion safety. However, it causes false positives because nearly one third of the HIV positive results were not confirmed by alternative tests. It is also possible to blame the lower sensitivity and specificity of these alternative tests to confirm HIV positivity despite their common use in SSA.

Taking into account all the tests carried out to confirm a blood donor screened positive for HIV and the costs incurred, it is appropriate to question the advisability of using viral nucleic acid testing (VNAT) to confirm positivity for HIV and other viruses instead of alternative tests. Confirmatory tests are mandatory for purposes of donor notification, counselling and possible actions to be taken regarding previous blood donations of the donor. Indeed, it is possible to use VNAT either for individual blood donations or by a strategy of ‘pooling’ 5 to 10 samples of donated blood^36^ screened positive by the chemiluminescent technique. Thus, only positive pools would be subjected to individual VNAT. This strategy, in addition to its reliability as formal proof of the presence of HIV in one or more donations, reduces the costs of confirming positivity of viruses in blood donors. In summary, in order to improve transfusion safety at a reasonable cost, screening by chemiluminescence can also be combined with confirmation by molecular viral genotyping using a pooling strategy. However, as discrepancies between the three tests are generally of samples with a URL value close to the cutoff, it would also be possible to pool 5 to 10 of these samples and only test these pools by VNAT. A reduced number of blood donations would be genotyped and these donations could be tested individually by molecular genetics. Notwithstanding, if resources are available, it is better to perform VNAT in individual blood donations instead of by pooling samples.

The goal in the transfusion process is to assure the availability and safety of the blood supply avoiding TTIs. In several countries, blood donation screening uses a viral component detection (HBsAg for HBV and the p24 antigen for HIV), or the antibody resulting from immune response such as anti-HIV and anti-HCV. The goal is to discard the correct blood donations with subsequent donor deferral or exclusion. A window period exists corresponding to a latent period of immunosilence shortly after infection where infection markers are undetectable, no matter how sensitive the laboratory screening and confirmatory assays are. Indeed, window periods are longer for antigen and antibody testing (42 days for HBsAg, 21 days for anti-HIV and 60 days for anti-HCV) than for VNAT. According to WHO guidelines the window periods for VNAT are 8, 27 and 5 days for HIV, HBV and HCV, respectively.^37^ These window periods increase slightly (11, 37 and 7 days for HIV, HBV and HCV, respectively) when testing small pools of blood donations.^37^

In SSA, the youthfulness of the blood donor population compared to the African population is a constant figure in most of the Africa countries south of the Sahara with an average age (∼ 30 years) generally lower than that observed in European countries.^38^, ^39^, ^40^ The difference in sex observed in donors can be explained in a large part by contraindications of women to donate blood. In SSA, a high endemicity (between 12 and 14 %) of HBV among blood donors^41^^,^^42^ (Figure 1) is found, predominantly in men (Figure 2) and in the 20- to 30-year age group (Figure 3). This not only raises questions about susceptibility to infection between the genders and differences in immune response to infections, but also the high participation of men as blood donors.^43^ Of course, it might also be related to sexual behavior in this country and all the related traditional and religious beliefs.^6^ However, HBV seroprevalence in the Maghreb region is lower than those in SSA.^31^^,^^44^ Unlike HBV, the seroprevalence of HCV in this study is lower than that found in other SSA countries.^42^^,^^45^^,^^46^ This higher prevalence of HCV compared to HIV poses a real public health problem. In fact, in this study we observed a prevalence of HIV significantly lower than the rates reported in other SSA countries^42^^,^^46^, ^47^, ^48^ but higher than those of Morocco^31^^,^^44^ and France.^49^

## Conclusion

This study updates the figures related to TTIs in blood donations in a SSA country, shows the advantages and limits of tests and also a strategic way to include chemiluminescence technique to screen blood donors. Despite the high sensitivity and specificity, microparticulate chemiluminescent immunoassay techniques can lead to false positives and also the viral window period problem must be remember. It might therefore be necessary to consider strategies to combine chemiluminescent technique with other serological tests and also screening using VNAT either of individual blood donation or using a pooling strategy as a confirmatory test.

## Conflicts of interest

The authors declare that they have no competing interest.

## Appendix Supplementary materials

Image, application 1
