# Supplementary material for: Improvement of blood transfusion safety using the chemiluminescence technique for viral marker screening of blood donors in sub Saharan Africa
Source: Hematol Transfus Cell Ther. 2024 Sep 7;46(Suppl 5):S72–9. doi: 10.1016/j.htct.2024.04.120 (PMC11670564; doi:10.1016/j.htct.2024.04.120)

**Figure S1:** Proposed algorithm of viral marker screenings in countries with limited resources such as sub-Saharan Africa using the chemiluminescent technique


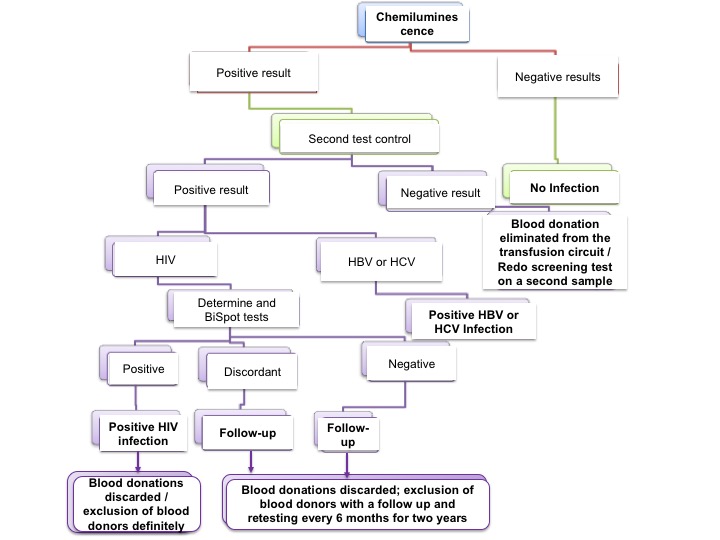

Supplement: Supplementary file 1 [file mmc1.docx]
